# The Double-Faced Role of Nitric Oxide and Reactive Oxygen Species in Solid Tumors

**DOI:** 10.3390/antiox9050374

**Published:** 2020-04-30

**Authors:** Sanja Mijatović, Ana Savić-Radojević, Marija Plješa-Ercegovac, Tatjana Simić, Ferdinando Nicoletti, Danijela Maksimović-Ivanić

**Affiliations:** 1 Department of Immunology, Institute for Biological Research”Siniša Stanković” National Institute of Republic of Serbia, University of Belgrade, Bulevar despota Stefana 142, 11060 Belgrade, Serbia; sanjamama@ibiss.bg.ac.rs (S.M.); nelamax@ibiss.bg.ac.rs (D.M.-I.); 2 Institute of Medical and Clinical Biochemistry, Faculty of Medicine, University of Belgrade, 11000 Belgrade, Serbia; ana.savic-radojevic@med.bg.ac.rs (A.S.-R.); m.pljesa.ercegovac@gmail.com (M.P.-E.); tatjana.simic@med.bg.ac.rs (T.S.); 3 Department of Biomedical and Biotechnological Sciences, University of Catania, 95123 Catania, Italy

**Keywords:** nitric oxide, reactive oxygen species, cancer therapy

## Abstract

Disturbed redox homeostasis represents a hallmark of cancer phenotypes, affecting cellular metabolism and redox signaling. Since reactive oxygen and nitrogen species (ROS/RNS) are involved in regulation of proliferation and apoptosis, they may play a double-faced role in cancer, entailing protumorigenic and tumor-suppressing effects in early and later stages, respectively. In addition, ROS and RNS impact the activity and communication of all tumor constituents, mediating their reprogramming from anti- to protumorigenic phenotypes, and vice versa. An important role in this dichotomic action is played by the variable amounts of O_2_ in the tumor microenvironment, which dictates the ultimate outcome of the influence of ROS/RNS on carcinogenesis. Moreover, ROS/RNS levels remarkably influence the cancer response to therapy. The relevance of ROS/RNS signaling in solid tumors is witnessed by the emergence of novel targeted treatments of solid tumors with compounds that target ROS/RNS action and production, such as tyrosine kinase inhibitors and monoclonal antibodies, which might contribute to the complexity of redox regulation in cancer. Prospectively, the dual role of ROS/RNS in the different stages of tumorigenesis through different impact on oxidation and nitrosylation may also allow development of tailored diagnostic and therapeutic approaches.

## 1. Introduction

In the highly sophisticated network of biological processes, certain molecules might have a dual role, depending on the context and their activity as a part of complex intra- and intercellular communication pathways. Some of them, such as reactive species, are involved in the maintenance of regular physiological settings, but in pathophysiological conditions they may become pathogenic effectors of cell damage and destruction, and contributors to disease development. For example, it is well known that impaired redox homeostasis, in association with significant metabolic shift, is one of the key determinants of malignant phenotypes.

Disturbance of homeostasis starting from the single-cell level transmits and amplifies from the surrounding area toward the whole organism. Cancer cells have the capacity to expresses different metabolic phenotypes, ranging from glycolysis to increased mitochondrial respiration, as an adaptive mechanism to immediate or chronic modifications of both extracellular and intracellular conditions. According to the fourth principle of the redox code postulated by Jones and Sies (2015), an adaptive redox network is necessary to preserve cellular homeostasis in a changing environment, and if functionally impaired, contributes to disease [1]. 

Indeed, it has been shown that oxygenation, glucose availability, and growth factors significantly affect intracellular reactive oxygen species (ROS) and nitric oxide (NO) levels, which in turn contributes to regulation of downstream signaling pathways. By modifying their metabolic phenotype, the cancer cells maintain steady-state ROS and reactive nitrogen species (RNS) levels within a narrow range, which allows them to increase growth and invasion, while limiting their apoptotic propensity [2,3]. Reactive species cannot be regarded as a single entity, since they are produced under different conditions and they all possess specific chemical properties [4,5]. They primarily comprise reactive oxygen and nitrogen species, but also sulfuric, chlorine, and bromine reactive species [5,6]. 

These molecules are produced as a result of aerobic metabolism, which is usually beneficial but is endowed with potential cellular toxicity at higher concentrations. It is generally accepted that at physiologically low concentrations, these molecules regulate a number of intracellular events, such as regulation of enzyme activity, post-translational modifications of newly synthesized proteins, signal transduction, regulation of gene expression, as well as regulation of apoptosis [6]. 

The aim of this review is to highlight the best-characterized aspects of the dichotomic role played from the ROS/RNS pathway in the regulation of solid tumors and the possible translation of these concepts to the clinical setting. Efforts will also be made to highlight the emergence of ROS/RNS tailored theragnostic approaches to be considered during specific stages of the tumor development.

## 2. The Double-Faced Role of ROS/RNS in Cancer

In physiological conditions, the role of ROS is preferentially directed towards redox signaling rather than oxidative damage to all types of macromolecules, including proteins, lipids, and DNA [7,8]. By definition, ROS/RNS comprise both free radicals, containing one or more unpaired electrons, such as superoxide (O_2_^•^), hydroxyl- (^•^OH), nitric oxide, alkoxyl (RO^•^), or peroxyl-(ROO^•^) radicals, along with non-radical ROS, which include hydrogen peroxide (H_2_O_2_), organic hydroperoxides (ROOH), and hypochloride (HOCl). The most reactive free radical, ^•^OH is highly reactive towards DNA and can activate certain oncogenes, such as K-Ras. Superoxide-stimulated cellular damage is also due to ^•^OH production via the Haber–Weiss reaction [9]. 

Among different ROS, H_2_O_2_ has emerged as a major redox metabolite, which is effective in redox sensing, signaling, and redox regulation [10,11]. H_2_O_2_ is recognized as a second messenger in several growth-factor-induced signaling cascades. It modulates the activation of the transcription factors activating protein-1 (AP-1), nuclear factor erythroid 2-related factor 2 (Nrf2), cAMP response element-binding protein (CREB), hypoxia-inducible factor α (HIF-1α), p53, and nuclear factor-κB (NF-κB), as well as signaling for epithelial–mesenchymal transition (EMT) [10]. Increased amounts of H_2_O_2_ may decide between the promotion or suppression of carcinogenesis in a dichotomic fashion [12], depending on the levels and the subcellular location of increased H_2_O_2_. While high amounts of H_2_O_2_ at the cell surface usually induce the activation of the cell cycle, a high H_2_O_2_ concentration in the mitochondrial compartment might inhibit cell cycle progression. Furthermore, various cancer cells are stimulated to undergo cell division at low H_2_O_2_ concentrations, while higher concentrations result in cell growth inhibition and even cell death. Thus, a predominant increase in O_2_^•^ (oncogenic ROS) supports cell survival and promotes oncogenesis, whereas a shift in favor of H_2_O_2_ (onco-suppressor) induces cell death signaling [13]. Hence, H_2_O_2_ can induce either cell proliferation or cell death, depending on its levels, with specific thresholds in specific cell types [12]. By using H_2_O_2_ as a thiol oxidant, specific protein cysteines function as redox switches, making this ROS essential for positioning the set point of the redox proteome [14].

## 3. ROS-Induced Genetic and Epigenetic Instability in Cancer Cells

**Figure 1:**
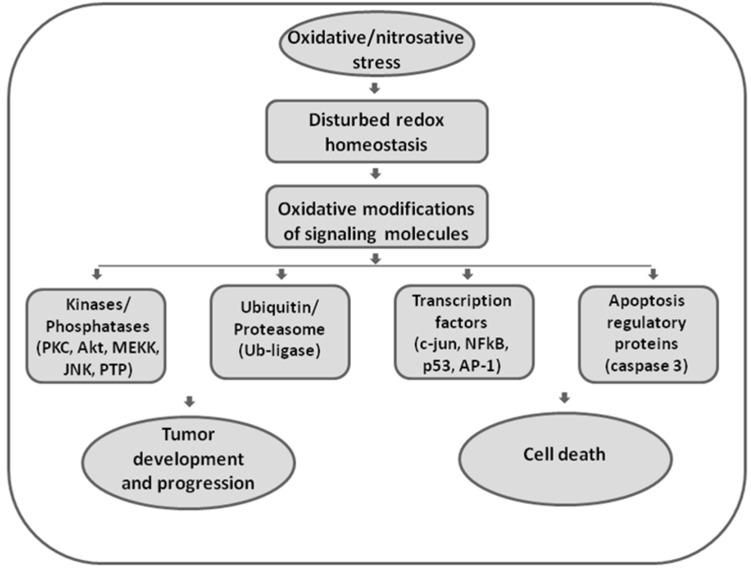
The role of oxidative and nitrosative stress in redox signaling. Disturbed redox homeostasis represents a hallmark of malignant phenotypes, affecting redox signaling in cancer cells. Reactive oxygen and nitrogen species (ROS/RNS) are effective in redox signaling and regulation by affecting the activity of redox-sensitive kinases and phosphatases, enzymes involved in ubiquitin and proteasomal degradation, transcription factors, and executor apoptotic molecules. Being involved in both regulation of proliferation and apoptosis, ROS/RNS seem to have double-faced roles in cancer.

Elevated ROS can either trigger apoptotic mechanisms or directly impair and damage cellular macromolecules (Figure 1).

The extent of oxidative damage of macromolecules depends on both ROS intracellular concentration and on the pro-oxidant–antioxidant balance, which is altered in numerous disorders, including cancer [7,15,16]. Indeed, increased ROS production and consequential oxidative stress represent hallmarks of carcinogenesis [6,7]. 

Oxidative stress and ROS accumulation might induce genetic and epigenetic instability in cancer cells [17] by impairing cellular repair mechanisms, thus causing DNA damage and mutations, and by affecting DNA methylation and demethylation, with consequential modification of the overall methylome [18,19], as well as indirect modulation of the activity of histone-modifying enzymes [20]. 

The main targets of oxidative damage are the CpG islands, as the oxidation of the methylated cytosine within CpG repeats produces hydroxy-methyl-cytosine (OH-mC), which initiates demethylation and may provoke further loss of epigenetic markers in some tumors, with consequential emergence of inappropriate transcription. In addition, the oxidation of either methyl-cytosine or guanosine (forming 8-oxo-guanosine) within properly methylated CpG islands causes a loss of inhibition for binding of transcription factors, leading to loss of epigenetic regulation [17,18,19]. Together with histone lysine methylation, the methylation of CpGs also regulates chromatin, whereby extensive methylation is followed by chromatin compaction, generating a transcriptionally silent state. By doing so, oxidative stress may also affect the epigenetic regulation at the histone, histone modifier, and chromatin structure levels [21].

There are multiple ways in which ROS can contribute to both tumor development and progression, including regulation of cellular proliferation, apoptosis, tissue invasion, angiogenesis, and metastasis. As recently suggested by Assi (2017), since ROS are involved in both the regulation of proliferation and apoptosis, they may play a double-faced role in different stages of the disease, entailing pro-oncogenic effects in the early stages and potential tumor suppressor effects in the late stages of cancer [21].

## 4. Pleiotropic Role of Nitric Oxide in Cancer

Nitric oxide (NO) is included as a universal signal in diverse biosystems, and plays a key role in communication both inside the cell and between the cell and the environment [22]. This feature was developed through evolution and was influenced by many factors. Due to its high chemical reactivity, the list of target molecules that may undergo significant biological and functional modifications in response to NO is very long [22]. The biological activities of NO are realized through cyclin guanosine monophosphate (cGMP)-dependent and cGMP-independent routes. The soluble form of guanylyl cyclase is the unique known “receptor” for NO. After the binding of NO to heme, which contains the GMP subunit, the enzyme guanylyl cyclase develops a cyclic configuration and becomes functionally competent in order to activate cGMP-dependent kinases, further transducing numerous signals through a cascade of phosphorylation of different proteins [23]. On the other hand, non-canonical cGMP-independent NO signaling is mainly based on *S*-nitrosylation of different target proteins, as well as other organic compounds [24], which is realized through NO covalent binding to alkyl sulfur atoms, without the support of enzymes. This chemical modification alters the protein function, stability, location, and protein–protein interactions [25]. Additionally, NO can be metabolized into other RNS, showing the dominant role of peroxynitrites in both physiological and pathophysiological backgrounds [26]. NO-mediated transformations of some of these target molecules may further affect gene transcription, making NO an indirect regulator of gene function [27]. This complexity clearly limits our ability to predict how all signals affected by NO can be translated into integrated and very specific outputs. 

**Figure 2:**
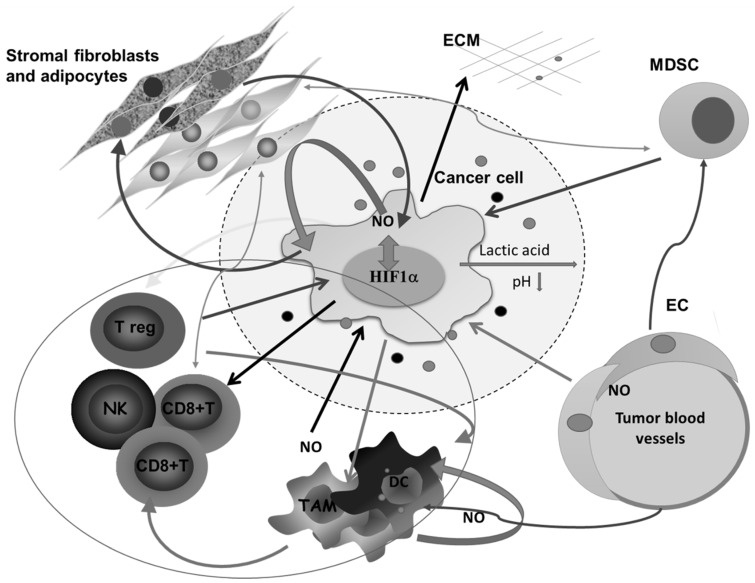
Complex intercellular communication between tumor cells and other members of the microenvironment in a solid tumor. O_2_ deficiency due to the compromised blood supply provokes the expression of HIF1α- and HIF1α-related genes involved in regulating the synthesis of nitric oxide (NO), glycolysis, and angiogenesis, and remodeling of the extracellular matrix. Lactic acid accumulation decreases pH in the surrounding area. Additionally, together with different molecules secreted by tumor cells, lactic acid affects macrophages (Mfs), dendritic cells (DCs), and cytotoxic lymphocytes, and potentiates accumulation of regulatory T cells (Tregs) and myeloid-derived suppressor cells (MDSCs). Tumor-associated macrophages (TAMs) express several M2-associated protumor functions, including promotion of angiogenesis, further matrix remodeling, and suppression of adaptive immunity. Stromal adipocytes and tumor cells have a symbiotic relationship mediated by NO. Cytotoxic lymphocytes, as well as MDSCs, in the absence of glucose supply, obtain energy from lipid storage by communicating with adipocytes. ECM—extracellular matrix, NK— natural killer cells, EC—endothelial cells.

All this is augmented in solid malignancies because of the heterogeneous nature of the tumor tissue, which consists of tumor cells in different stage of differentiation or trans-differentiation; the presence of stem cells, vascular-like endothelial cells, cancer-associated fibroblast (CAF), normal tissue counterparts, and immune cells; and the matrix that connects all these constituents into a unique network (Figure 2). 

NO can be produced from tumor cells and non-malignant cells in the vicinity of a tumor. The connections between chronic inflammation, cancer development, and NO synthesis are well documented [28]. On the other hand, the role of this molecule in early stages of tumor progression is probably not essential, however it is in the upcoming hyper-proliferative phase [28]. 

## 5. High Steady-State ROS Levels in Tumor Cells

Recent evidence has suggested that the high steady-state ROS levels in cancer cells may play an important role in tumor progression. Indeed, inhibition of either ROS production or increase in antioxidant capacity results in diminished proliferation of cancer cells, both in vitro and in vivo [6,7,29]. 

Different factors involved in tumor progression, including hypoxia, activated Ras, and a variety of growth factors—especially epidermal growth factor (EGF), fibroblast growth factor (FGF), platelet growth factor (PDGF), and TNF-α—increase intracellular ROS by activating the primarily membrane-bound family of the NADPH oxidase (NOX) enzymes [30,31]. Furthermore, moderate mitochondrial ROS can also favor tumor growth and more invasive cells show elevated mitochondrial respiration [32,33,34]. The relevance of mitochondrial ROS for tumor growth and migration is consistent with the fact that neutralization of ROS by pharmacological manipulation of key antioxidant enzymes, such as catalase (CAT) overexpression or addition of superoxide dismutase 2 (SOD2) mimetics, negatively affects both processes [33,35,36]. The fact that a dysfunctional electron transport chain may be beneficial in promoting migration and invasion [34,37] suggests that the underlying mechanism is due to ROS rather than ATP production. Indeed, increased ROS production followed by advanced metastatic potential of cancer cells with a dysfunctional electron transport chain has been demonstrated in various studies [34,37,38].

The physiological attempt to counteract ROS accumulation in transforming cells is to upregulate antioxidant systems. In an effort to oppose the effects of ROS, cancer cells induce the expression of different antioxidant enzymes, including glutamate cysteine ligase (GCL), glutathione S-transferase (GST), glutathione peroxidase (GPX), SOD, CAT, and thioredoxin (Trx) [39,40,41]. Increased activity of GCL, a key regulatory enzyme of glutathione (GSH) synthesis, is the strategy used by many tumors to increase the content of this primary cellular antioxidant, which buffers ROS. The GST superfamily contains detoxification enzymes, which catalyze the conjugation of GSH with a wide variety of xenobiotics, including oxidative stress products. These enzymes are considered relevant in both cancer development and progression. Thus, in general, over-expression of GST class P1 seems to be a hallmark of proliferating cells in many solid tumors [42]. In addition, the increased expression of antioxidant and detoxifying enzymes may correlate with malignant potential of different solid tumors [40,43]. This is of particular relevance for cancer cells, in which ROS production is important for the alteration of normal cells to cancer cells on one hand, but also might promote cancer cell death on the other hand [20,44]. Indeed, inhibition of antioxidant pathways compromises the cancer’s ability to handle oxidative stress and results in cell death [45,46]. 

Regulators of the antioxidant response also control metabolic phenotypes. Apart from increasing GSH content, the pentose phosphate pathway, which synthesizes NADPH, is also intensified in cancer cells, as a mechanism used to fight oxidative stress resulting inincreased survival. Therefore, in contrast to precancerous conditions—in which antioxidant defense is decreased and ROS are able to induce DNA damage, mutations, and tumor development—in the late stages of carcinogenesis, increased antioxidant activity counteracts excessive oxidative damage, thus enabling cancer cells to escape apoptosis [6,7,47]. Indeed, it seems that the vast majority of solid tumors have higher steady-state ROS levels [2,48,49]. 

This new redox balance enables cancer cells to develop resistance to ROS, further resulting in cellular adaptation and proliferation, simultaneously enabling their escape from oxidative damage, as suggested by Sosa et al. [6].

## 6. Multiple Roles of ROS/RNS in Cancer Proliferation

Intensive cell division rates in cancer require intensive anabolic processes, which demand extreme ingredient and energy acquirement from degradation of nutrients. Soon after initiation and early progression of the tumor, the density of the blood vascular network rapidly becomes too low to support the perfusion of the whole tumor. As a consequence, some tumor cells are placed far away from existing blood vessels to receive sufficient O_2_ and glucose supply, resulting in the presence of necrotic areas within tumors. In order to adapt to the new oxygen deficiency conditions, tumors develop their own blood vessel network, which is structurally and functionally much different than in healthy areas [50]. The disordered and poorly hierarchical structure of the tumor blood vessel network further causes irregular tumor tissue perfusion and variable oxygenation, with periodical replacement of hypoxic and normoxic phases. All these aspects are reflected in dramatic changes in cell signal transduction, cell metabolism, gene expression, and overall changes in cell behavior at individual and collective levels. 

Higher metabolic activity and increased energy demands, accompanied with enhanced ROS production, further affect the growth process. In the context of high division rate, there is a shift in glucose metabolism from glycolysis to the pentose phosphate pathway, which is necessary for nucleotide biosynthesis to prevail (128). Additionally, the production of NADPH is required to maintain the reduced state of the most abundant non-enzymatic antioxidant, GSH [6]. 

Once the cells start their transformation into a more aggressive phenotype, there are multiple signaling molecules targeted by ROS, which are activated (such as transcription factor Smad, matrix-metalloproteinases (MMPs), hepatocyte growth factor receptor, transforming growth factor β-activated kinase, etc.) and further allow the cells to obtain the migratory properties and translocate via the bloodstream to different parts of the organism [51,52,53]. Based on evidence on the specific localization of NOX enzymes in membrane protrusion structures (invadopodia), their role in facilitating the invasion process has also been proposed [54]. In fact, many of the effects of NOX-induced ROS signaling may be mediated by activation of Src kinase, including the activation of matrix-degrading MMPs [55]. Therefore, different ROS-associated signaling pathways, including integrin-mediated mitogen-activated protein kinase (MAPK) signaling, protein tyrosine phosphatases, and p21-activated kinase 1, are involved in the EMT process [6,51,52]. Regarding the role of oxidative stress in proliferation, there is a number of signaling cascades that are affected, such as kelch-like protein 19 (Keap1) and Nrf2, as the master regulators of the antioxidant response, but also Ras, Raf, and numerous MAPK [6,55,56].

Another important process in tumor growth and metastasis is angiogenesis. Among proangiogenic factors produced by tumor cells, vascular endothelial growth factor (VEGF) is recognized as the key regulatory protein, which is upregulated in majority of human cancers [57,58]. Interestingly, apart from growth factors and cytokines, hypoxic conditions and increased ROS production are shown to contribute to this increase [6]. Hypoxic conditions are frequently generated in cancer cells due to high levels of proliferation. In order to avoid this, in cancer cells HIF1α and HIF2α escape the proteasome-mediated degradation, which normally happens under normoxic conditions [59,60]. This accumulation of HIF1α proteins results in downstream overexpression of HIF target genes, which regulate angiogenesis, proliferation, cell migration and invasion, glycolysis, and survival [61].

## 7. NO-Related Intracellular Hypoxia Provokes Tumor Progression

**Figure 3:**
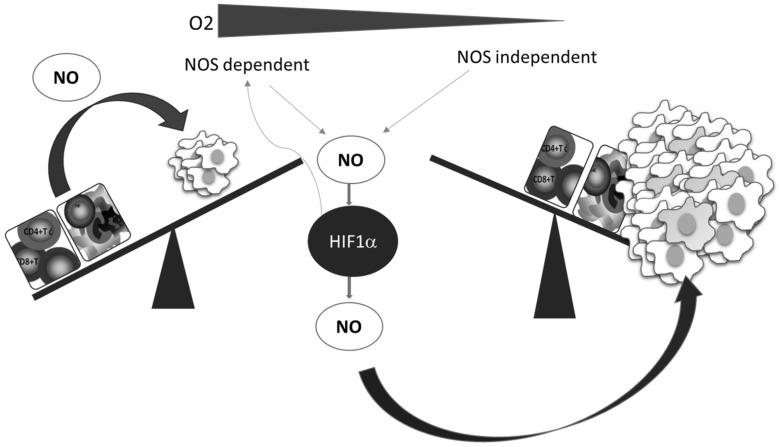
Oxygen deficiency and HIF-1–NO interplay favors tumor progression and immune escape. Decreased oxygen supply potentiates involvement of NO and HIF-1α in the regulation of main cellular processes. These conditions preferentially support tumor cell proliferation and protumor activities in innate and tumor-specific responses.

A better understanding of the relationship between NO and HIF-1, which is the key mediator of the cellular hypoxic response, underlines the importance and involvement of NO, as well as of the RNS produced in reactions between NO and ROS. Numerous pathophysiological processes in cancer are regulated by the HIF-1–NO interplay (Figure 3) [62].

Additionally, while NO regulates the activity of HIF-1, the hypoxic environment remarkably affects the synthesis of NO. Two main constitutively expressed intracellular NO producers, namely endothelial (eNOS) and neuronal (nNOS), together with the inducible enzyme (iNOS), catalyze l-arginine-dependent NO synthesis in the presence of NAD(P)H and oxygen [63]. Although the exact level of oxygen necessary for this reaction is unclear, it is known that the arginine pathway for NO synthesis is abrogated in oxygen-depleted environments [64]. Instead of NOS mediated NO synthesis, the alternative NOS-independent pathway of NO production becomes dominant under these circumstances (Figure 3). This NOS-independent route of NO production is enabled by a few heme-containing proteins [28,65], such as hemoglobin, myoglobin, xanthine oxidase, cytochrome P450, cytochrome c oxidase, and cytochrome c [65]. It should be noted that although most studies confirmed that NO mainly stabilizes HIF-1α, there are some contradictory data, which may be due to diverse conditions and experimental settings and the consequence of the highly sensitive and complex network involved in NO intracellular activities. The quality of NO and HIF-1 interactions varies depending on the concentration, time, and duration of NO exposure, but also the level of surrounding oxygen [28,62]. 

The main molecular mechanism of NO-mediated HIF-1 protection from proteasomal degradation is nitrosylation of certain cysteine residues [66]. Moreover, NO stabilizes HIF-1α by S-nitrosylation of Ras-Cys118, affecting phosphoinositide-3-kinase–protein kinase B (PI3K/Akt) signaling. This further amplifies NO availability by promoting NOS expression [67]. In addition to nitrosylation, as a leading post-translational regulatory mechanism of HIF-1 availability, the transcription factor activity can be modulated by acetylation, which is indirectly dependent on NO [48]. Altogether, in hypoxic conditions, which is typical for solid cancers, nitrosylation is among those key post-translational modifications that are responsible for regulation of signaling events in the cells. Hence, NO may be a pivotal molecule for cancer cell signal transduction in high-grade and aggressive forms of solid cancers with regions of chronic or cyclic hypoxia. The cellular NO pool is continuously recycled using NO produced both by NOS-independent and NOS-dependent pathways.

Tumor-promoting features of S-nitrosylation, as a main post-translational modification triggered by NO, are not limited only to proteins involved in different pathways engaged to enable tumor proliferation. An appropriate, rational, and safe mode of energy uptake and consumption is extended to lipid storage in tumor tissues or the surrounding area, supporting tumor growth. It was found that abnormal S-nitrosylation in the presence of a high intracellular concentration of NO increases the adipogenesis and amplifies the number of adipocytes, which serve as tumor lipid storage sites [68,69]. It is extremely important to underline the metabolic crosstalk between the stromal adipose cells and tumor cells, as upon the uptake of arginine released from adipose cells the cancer cells start to produce NO, using it for vital functions and division. The metabolic product of this synthesis, citrulline, is subsequently consumed by adipose cells, increasing the lipid storage. This loop illustrates the perfect NO-mediated symbiotic relationship between cancer and stromal adipocytes [69] (Figure 2).

**Figure 4:**
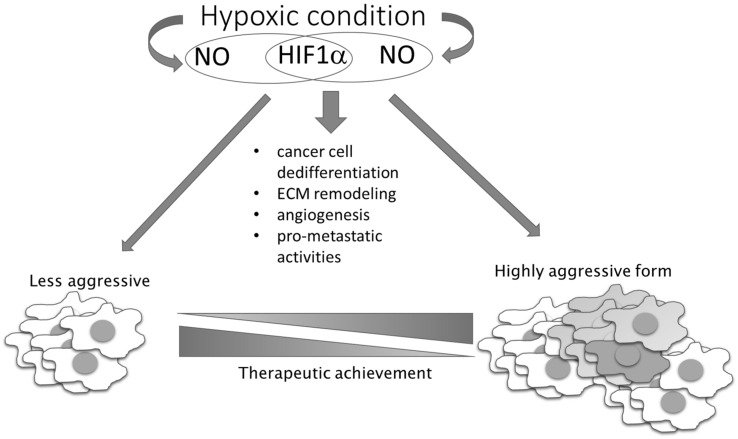
NO–HIF-1 input in tumor progression and therapeutic achievement. NO together with HIF-1 influences the main processes involved in the conversion of less-aggressive toward high-aggressive phenotype. HIF-1 regulates the expression of genes responsible for stem phenotype establishment, remodeling of the extracellular matrix (ECM), angiogenesis, and NO synthesis, while NO influences HIF-1 stability.

In contrast to these pro-oncogenic roles of NO under conditions of unstable oxygen supply in tumor tissue, there are also NO-mediated activities based on the same mechanism that exhibit tumor suppressing properties. The presence of NO can prevent ROS elimination by GSH, as S-nitrosylation of GSH results in generation of inactive S-nitrosoglutathione with consequential accumulation of ROS in highly glycolytic and hypoxic cells, where it promotes apoptotic cell death [70]. In addition to this, higher concentrations of NO and peroxynitrite produced in interplay of NO and ROS can directly kill tumor cells. This observation has propelled numerous therapeutic approaches, entailing use of exogenous NO donors as chemotherapeutic agents. Hence, we and others have generated and studied different NO derivatives of aspirin, antiretroviral protease inhibitors (saquinavir, lopinavir, and ritonavir) [29,71,72,73,74,75,76,77,78], and histone deacetylase 1 (HDAC1) inhibitor [79]. Although in vitro and in vivo preclinical data have convergently shown a substantially higher chemotherapeutic potency of the NO-hybridized compound as compared to the parental drug, none of these NO-hybridized molecules have been advanced to the clinical stage for the treatment of cancer. It is also worth noting that double-hybridized molecules are emerging, including compounds with simultaneous capacity to release NO and the other pleiotropic endogenous gas H2S, which are attracting further interest due to the possible modulation of endogenous gases as chemotherapeutic tools [80]. Additional studies on this series of compounds are needed to ascertain their feasible translation in clinical settings. However, critical issues relating to the targeted delivery, dynamics, and concentration of released NO that are related to the complex and pleiotropic nature of NO in cell physiology needs to be clarified to significantly advance the use of NO donors, and possibly combined NO and H_2_S donors, in clinical settings [29,71,72,73,74,75,76,77,78,80,81,82,83,84,85,86]. As a bystander product of cellular metabolism, NO has also been described as being very important in metabolic crosstalk between cancer cells and other members of the cancer microenvironment. Disturbed oxidative phosphorylation and abrogated mitochondrial respiration in relation to NO reaction with complex IV of the electron transport chain leads to a metabolic hypoxia state [87]. NO can promote tumor aggressiveness and development of a chemotherapy-resistant phenotype, simulating hypoxic conditions (even in the presence of oxygen), promoting glycolysis, and diminishing mitochondrial respiration (Figure 4) [88].

**Figure 5:**
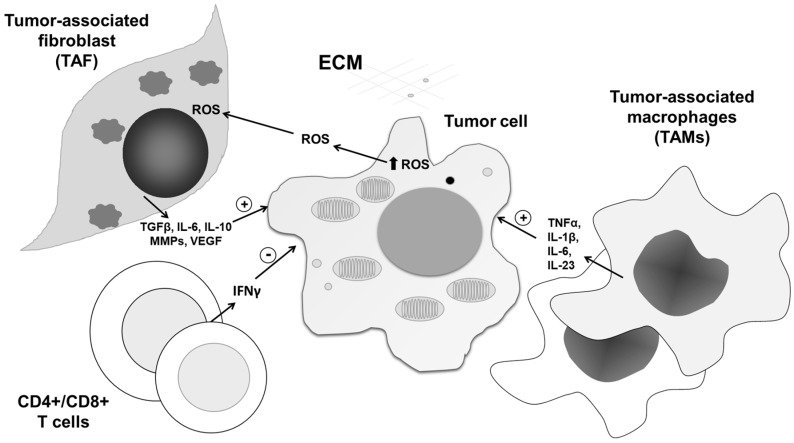
Role of vascular-derived HIF-1α and NO production in tumor dissemination. Endothelial HIF-1 α triggers the expression of iNOS, and through NO production affects the entering of tumor cells into circulation, as well as their dissemination into distant sites. ECM—extracellular matrix, ROS—reactive oxygen species, IL—interleukin, MMP—matrix metalloproteinases, VEGF—vascular endothelial growth factor, TGF-β— transforming growth factor beta, IFN-γ— interferon- γ.

NO-related intracellular hypoxia provokes tumor progression through multiple pathways, including through increasing the glycolitic process and augmented consumption of glutamate in the TCA cycle. Aerobic glycolysis leads to decreased pH in the microenvironment as a consequence of lactate accumulation from the creation of an acidic environment, which downregulates activated T cells [89]. The same condition provokes angiogenesis, as well as M2 polarization of macrophages [90]. In low-differentiated, high-grade tumors, the M2 phenotype of macrophages is at least partly responsible for an insufficient chemotherapeutic response (Figure 2) [91]. In addition to the cytoprotective role resulting from the establishment of the intracellular antiapoptotic profile, NO indirectly limits chemotherapeutic efficacy in poorly differentiated tumors, at least partly through macrophage polarization toward M2. These relations between NO and tumor cell metabolism are preferentially found in invasive, high-grade tumors. Concordantly, and primarily through its tight interaction with endothelial HIF-1, NO is recognized as an important regulator of oncogenic pathways controlling tumor spreading, including S-nitrosylation of adherent junction complexes, promotion of EMT, invasion and dissemination through changes in tumor cell adhesion to endothelial cells, intra- and extravasation, as well as endothelial cell permeability [92,93] (Figure 5).

## 8. ROS and RNS Effects in the Tumor Microenvironment

The presence of inflammatory cells in cancer tissue shows their attempts to eliminate the transformed cells. Although this can result in eradication of certain tumors, it may also paradoxically result in promotion of tumor growth. As byproducts of cellular metabolism, ROS are crucial weapons of the innate immune response in the first line of elimination of pathogen and neoplastic cells. The moment at which the cancer cells establish ROS-resistant phenotypes and utilize them for their own maintenance and propagation is influenced by numerous factors [94].

**Figure 6:**
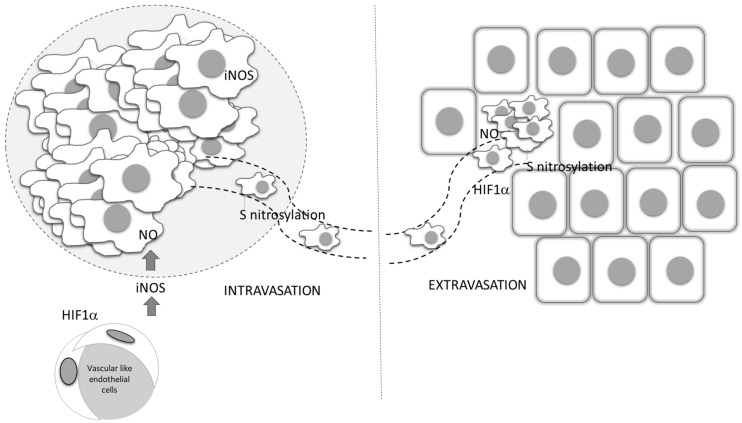
The roles of ROS/RNS in tumor tissue network interplay. In addition to affecting cancer cells, ROS/RNS impact the activity and communication of all constituents in the tumor tissue network, mediating their reprogramming from anti- to protumorigenic phenotypes and vice versa. After being released from tumor cells, ROS/RNS affect the tumor microenvironment, inducing release of cytokines, matrix metalloproteinases (MMPs), and signaling molecules from cancer-associated fibroblasts (TAFs), CD4+/CD8+ T cells, and tumor-associated macrophages (TAMs). In this way, the tumor microenvironment contributes to tumor progression in response to increased ROS/RNS levels.

Increasing evidence indicates that one of the leading processes involved in malignant cell adaptation to intracellular ROS is autophagy. This self-digestion process of removal of damaged organelles enables reuse of building molecules and promotes survival of cells in tumor tissue. Importantly, it was recently found that autophagy may support tumor growth, not only through recycling of diminished structures, but also as a recipient of information transduced by release of extracellular ROS in the tumor microenvironment [94]. Therefore, anti- and prosurvival roles of ROS in the tumor microenvironment can be copied into the dual roles of autophagy, defined by the type, concentration, and place of ROS generation. It was discovered that ROS can link tumor-associated fibroblasts (TAFs) and tumor cells through autophagy, providing a protumorigenic environment. Indeed, in a breast cancer xenograft model, HIF-1α-triggered autophagy in stromal cells is responsible for tumor progression [95]. With respect to intensive intrinsic autophagy, TAFs are preferentially resistant to ROS. Interestingly, communication between malignant cells and their neighborhood is partly realized through autophagy, as well as selective degradation of mitochondria by autophagy, known as mitophagy. This interplay is also very important as a source of anabolic blocks, as highly proliferative tumor tissue in hypoxic conditions is exhausted. Finally, the produced ROS further augments the malignant phenotype of TAF [96,97]. Consequently, TAFs further stimulate tumor growth and prevent immune response against cancer through the release of MMPs and several cytokines. Tumor growth is further stimulated by nearby senescent cells, which release proinflammatory cytokines and proteases into the tumor microenvironment [6] (Figure 6).

An acidic environment also stimulates the process of autophagy. This scenario is consistent with the expression of autophagy-related proteins in pre-invasive and invasive breast cancer [98].

Furthermore, acidosis and hypoxia strongly affect the accumulation, as well as the function of immune cells. Production of IFN-ƴ, IL-2, TNF-α, granzyme B, and perforin by NK cells, T cells, and monocytes is dramatically decreased, enabling establishment of anti-inflammatory and immunosuppressive environments [89,99]. Since proinflammatory cytokines are responsible for iron sequestration in macrophages and their ROS-mediated cytotoxic action, the perturbation of this process results in reduced capacity of immune cells to limit tumor growth. Thus, TAMs in the tumor microenvironment acquire protumorigenic and iron-donating phenotypes [100]. In addition to the importance of iron for tumor cell proliferation, its presence in the tumor microenvironment affects the H_2_O_2_-mediated antitumor immune response. Replacing of H_2_O_2_ via the ^·^OH generating Fenton reaction results in extracellularly produced ^·^OH, which due to its high reactivity, is unable to reach sensitive intracellular targets of tumor cells, abrogating this branch of cytotoxic activities of immune response [101]. 

Immune cells in the tumor microenvironment are also affected by all of these aspects, especially by abnormal blood supply, glucose restriction, and the acidic environment. Tumor-infiltrated lymphocytes (TILs) compete for glucose in the tumor niche, where they starve as a consequence of domination of tumor cell glucose uptake [102]. This glucose restriction can be overwhelmed by fatty acid catabolism of CD8+ TILs [103]. In competition for the lipid sources in the tumor mass with different potential consumers, such as TILs, tumor-associated myeloid-derived suppressor cells (MDSCs) enhance fatty acid uptake and oxidation in parallel with upregulated iNOS-mediated NO production and peroxinitrite generation, ultimately diminishing T cell proliferation [104]. Finally, both the function and survival of pivotal cells in nonspecific antitumor immune response, such as macrophages and tumor-dendritic cells, are critically connected with NO production. When NO production is augmented, oxidative phosphorylation and mitochondrial respiration are abolished in a manner similar to that observed in tumor cells. In macrophages, this restriction results in decreased IL-10-mediated immunosuppression and more stable M1 phenotype maintenance [105]. Concerning dendritic cells, metabolic hypoxia promoted by NO is essential for their activation and shaping of their immune function [106]. Under hypoxic conditions, the ability of dendritic cells to maturate and present antigen is abrogated. Additionally, they negatively regulate T cell function. All of these features are ascribed to tumor-derived VEGF [107], which is also closely connected with accumulation of myeloid-derived suppressor cells (MDSCs) and subsequent angiogenesis and dissemination.

In summary, diverse environmental factors, which contribute to increased ROS production at both the primary and metastatic sites, may affect tumor progression. Redox-sensitive signaling molecules involved in this adaptive process include HIF-1, ERK1/2, p53, and peroxisome proliferator-activated receptor-gamma coactivator-1 (PGC-1). Of these, HIF-1 and ERK1/2 are primarily activated in response to hypoxia, followed by increased ROS production [108]. HIF1α regulates the expression of genes involved in aerobic glycolysis (GLUT1, heksokinase 2), angiogenesis (VEGF), and the epithelial mesenchymal transition process (ZEB-1, MMP9). The opposite cancer metabolic phenotype is mediated by PGC-1, a central regulator of energy metabolism, which belongs to the family of transcriptional coactivators known as PPAR γ coactivators, and which is involved in synchronization between environmental stimuli and mitochondrial biogenesis and metabolic flux. Moreover, negative reciprocal regulation between HIF-1 and PGC-1 has been demonstrated in various investigations [49,109]. Thus, it has been shown that HIF1-mediated suppression of PGC-1α is critical for metabolic reprogramming in VHL-deficient clear cell renal cell carcinoma [109]. In defining appropriate metabolic response to ROS, the energy sensor AMP-activated protein kinase (AMPK) might represent one of the key molecular switches [110]. In that context, AMPK might determine the specific metabolic phenotype, from induction of oxidative phosphorylation by PGC-1 activation, glycolysis as a result of HIF-1 stimulation, or a more dormant phenotype by inhibiting the pleiotropic growth regulator, mTOR [111]. 

While moderate levels of ROS increase PGC-1 and oxidative phosphorylation, the effect of high ROS, together with altered environmental conditions such as starvation, might cause an inadequate metabolic response, and consequently increase autophagy [112]. In this context, autophagy contributes to inhibition of tumor growth and reduction of ROS production [113]. Moreover, in comparison to both primary and metastatic tumors, the aerobic metabolic phenotype is characterized by having a majority of circulating tumor cells [114]. Therefore, both glycolysis and upregulated antioxidants favor cancer cell survival under extreme conditions. It seems that the interplay between ROS, HIF-1, and Nrf2 has a pivotal role in this process [6,49].

Molecules that also contribute to angiogenesis via production of ROS and redox signaling pathway activation, including HIF1α induction, are NOX molecules, especially Nox1, Nox4, and Nox5 [115]. Thus, in highly proliferative cancer cells, regulation of ROS production seems to be crucial, especially considering the presence of oncogenic mutations, which promote aberrant metabolism and gene expression, simultaneously leading to increased ROS rates. 

## 9. NO in Initial and Acquired Resistance to Therapy 

**Figure 7:**
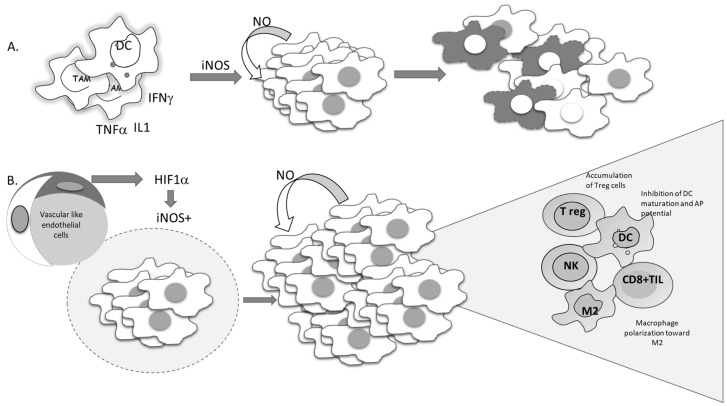
Conflicting outcomes of iNOS-produced NO in tumor cells. (**A**) Proinflammatory cytokines trigger the expression of iNOS in tumor cells, promoting NO-mediated cell suicidal activities. (**B**) Hypoxia-driven expression of iNOS-mediated tumor cell proliferation and re-education of the immune cells in the tumor microenvironment.

Current molecular targeted cancer treatments, in addition to affecting tumor cells, affect different constituents of the tumor microenvironment, such as immune, vascular, endothelial, and stromal cells, with the aim of switching all protumorigenic activities toward productive antitumor responses. Standard nonselective cytotoxic therapy is often used in conjunction with tailored molecular approaches [116]. For decades, the apoptosis of tumor cells has been seen as a desirable outcome of chemotherapeutic treatment, and development of resistance to chemotherapy has been associated with the development of an apoptotic-resistant phenotype. Since NO can suppress proteolytic activation, as well as the function of activated caspases through S-nitrosylation of cisteine residues in active enzyme sites, it can affect both receptor-dependent and -independent apoptosis in both normal and malignant phenotypes [15,117,118]. It is well documented that the intrinsic feature of malignant cells required to produce NO, mostly by constitutive expression of iNOS, is related to poor prognosis. Liao et al. recently reported a highly significant association between iNOS expression and disease outcome following overall, cause-specific, and disease-free survival [119]. According to results obtained through meta-analysis, all parameters were remarkably worse in patients with solid tumors expressing iNOS. Continuously released NO in cancer cells becomes necessary to maintain viability. The cytoprotective role of this molecule in the malignant cells is at least partly related to its ability to inactivate caspases. On the other hand, some data indicate that proinflammatory cytokines are able to promote suicidal iNOS expression in malignant cells, such as mouse fibrosarcoma L929 or rat astrocytoma C6. In these types of cell lines, endogenous NO provokes suicidal cellular activity, which is neutralized by specific iNOS inhibitors (Figure 7A) [120].

Several studies performed in recent years have suggested the importance of the signal responsible for the gene expression of iNOS. This point should be the most informative in predicting tumor cell survival. Namely, if iNOS expression is triggered by proinflammatory cytokines, tumor-cell-generated NO would serve as a host defender and executor of the program initiated by the immune system (Figure 7A).

Considering the heterogeneity of solid tumors, the source of NO production, together with its concentration and dynamic nature, appears to be of great relevance. Each type of cell in the tumor microenvironment can produce NO. If tumor cells constitutively express iNOS, NO production will most often support the cells own vitality and progression (Figure 7B). On the other hand, NO produced by immune cells in the tumor microenvironment represents one of the most powerful weapons for killing tumor cells. Endothelial NO is characterized by its basic task—to dilate the vessels. Taking advantages of these features, Xu et al. created tailored switchable NO-releasing nanoparticles (IPH-NO) comprised of photosensitizer (IR780), paclitaxel (PTX), and NO donor-S-nitrosated human serum albumin [121]. The photosensitizer increased tumor vascular permeability, promoting drug accumulation. Fast release of huge amounts of NO upon near-infrared light irradiation subsequently induced tumor cell death. IPH-NO also inhibited metastasis through EMT blockade. The final effect of the NO-mediated activity mentioned above will be defined by all factors in the tumor mass, including the specificity of tumor perfusion and the abnormal vascular network. When produced from tumor cells, NO promotes polarization of macrophages toward protumorigenic phenotype M2 (Figure 7B). In parallel, when NO is produced by macrophages by themselves, they retain their cytocidal profile (M1) [105].

New concepts indicate that activated and efficient caspases are not explicitly a sign of good therapeutic response and sensitivity to apoptosis [122]. Paradoxically, tumors with deficient caspase 3 seem to have better prognosis in comparison to those with conserved caspase 3 functions [85,123]. This may be related to the fact that caspases possess other functions in addition to regulation of cell death, including the maintenance of homeostasis in multicellular organisms. Caspases are equally employed by the death signals and signals involved in regeneration. They are the main messengers delegated to inform the neighbors that a dying cell needs to be replaced by a newborn cell, triggering the proliferation of progenitor cells [123]. Therefore, in high-grade tumors, poorly differentiated cells will divide in response to apoptosis in the surrounding area, explaining tumor repopulation after cytotoxic therapies. In this context, the ability of NO to affect caspases can reflect apoptosis-induced proliferation, decreasing the overall tumor progression, especially in response to certain cytocidal treatments. An alternative approach that bypasses the effects of compensatory proliferation and tumor repopulation and includes NO is the induction of the phenotype conversion of poorly differentiated cancer cells into more mature, less malignant forms [71,72,78]. NO-hybridized HIV inhibitors, namely lopinavir, saquinavir, and ritonavir, were able to decrease the malignant potential of glioma and melanoma cells through induction of differentiation, trans-differentiation, and senescence [72,77,78].

Although NO can mediate the development of chemotherapeutic resistance, extensive data confirm its potential to re-establish malignant cell sensitivity to immune-cell-mediated antitumor activities, as well as to make them sensitive to chemotherapy [75]. Elimination of Yin Yang 1 (YY1)-mediated repression of Fas and Death receptor 5 (DR5) genes is one of the main routes of immune sensitization triggered by conventional NO donating drugs. This can also be achieved with hybrid molecules modified by NO covalent attachment, such as the HIV inhibitor saquinavir [75]. Interestingly, NO-modified saquinavir strongly upregulated the functional DR5 receptor, even if the effect was not directly related to the amount of NO released from the molecule. Moreover, the same group of drugs promoted inhibition of NF-kB, leading to decreased expression of antiapoptotic genes and subsequent sensitization to apoptosis. Bonavida et al. found that NO interactions with the members of the antiapoptotic loop, consisting of NF-κB/Snail/YY1/RKIP/PTEN, in malignant cells is mainly responsible for their sensitization to the cytotoxic activities of immune cells, as well as re-establishment of susceptibility to chemotherapy [124].

## 10. ROS/RNS-Mediated Mechanisms of Targeted Cancer Therapy

Despite the fact that progression of solid tumors is frequently associated with disturbed redox homeostasis, potential effects of targeted cancer therapy, which also achieves a ROS-mediated mechanism of action, have rarely been systematically studied. Indeed, there is emerging evidence that at least some of these compounds, in addition to their primary specific antitumor roles, might also disrupt cellular redox homeostasis, usually in favor of oxidative distress, sometimes enabling even more reduced milieu. Still, it remains unknown how these redox perturbations impact tumor cell death, especially regarding the resistance of solid tumors. Based on the concept applied in the first review, which addressed ROS-mediated mechanisms of action of tyrosine kinase inhibitors and monoclonal antibodies, we reviewed the latest literature data on the most clinically relevant agents against epidermal growth factor receptor (EGFR), VEGFR, and human epidermal growth factor receptor (HER), for which substantial evidence exists that their application is associated with disruption of redox homeostasis [125] (Table 1). 

### 10.1. Free-Radical-Mediated Effects of Therapy Targeting VEGFR

Drugs targeting VEGFR are tyrosine kinase inhibitors (TKIs) that exhibit different kinase specificity [138]. Indeed, it has been shown that although different drugs sometimes share target specificity, they might differentially affect cancer redox homeostasis. Thus, multikinase inhibitors, such as sunitinib and sorafenib, inhibit proliferation and angiogenesis by blocking vascular endothelial growth factor receptors (VEGFR-2 and VEGFR-3), platelet-derived growth factor receptor-beta (PDGFR β), and RAF kinase, simultaneously influencing the redox state in opposite directions [139]. Antioxidant effects of sunitinib are accomplished by both upregulation of GSH content and inhibition of neuronal NOS activity [127,140]. On the contrary, when applied in combination with chloroquine, sunitinib leads to an increase in RNS and apoptosis via iNOS [141]. It is important to note that Nrf2–ARE signaling promotes the progression of solid tumors, while its inhibition enhances the sensitivity to sunitinib by arresting cells in the G0/G1 phase and increasing apoptosis in the tumor cell line [142]. On the other hand, sorafenib exhibits pro-oxidant effects by reducing the GSH pool [139]. Upregulated mitochondrial ROS production is mediated by mitochondrial dysfunction and an increase in superoxide anions [143]. In addition, in patients with hepatocellular carcinoma HCC treated with sorafenib, increased levels of advanced oxidation protein products were found, affecting their survival. Indeed, oxidative stress and apoptosis induced by sorafenib are influenced by fibroblast growth factor 19 (FGF19) and its receptor. It has been shown that FGF19, when overexpressed, inhibits the effect of sorafenib on ROS generation and apoptosis in HCC. On the other hand, loss of FGF19 or its receptor leads to a remarkable increase in sorafenib-induced ROS generation and apoptosis. Importantly, targeting of the FGF19–receptor axis by ponatinib, a third-generation inhibitor of chronic myeloid leukemia, overcomes the HCC resistance of sorafenib by enhancing ROS-associated apoptosis in sorafenib-treated HCC [144]. The other VEGFR-targeted drug, axitinab, induces oxidative DNA damage and exhibits immunomodulatory effects, leading to mitotic catastrophe and a cellular senescence program [42,145].

### 10.2. Free-Radical-Mediated Effects of EGFR-Targeted Therapy

The effects of several other TKIs, such as gefitinib, erlotinib, and afatinib, which are designed to target mutated epidermal growth factor receptor (EGFR) [8], are also associated with impaired redox homeostasis. Erlitinib is capable of inducing ROS-mediated apoptosis via activation of the c-Jun N-terminal kinase (JNK) pathway, which consequently leads to EGFR inhibition, which is blocked in the presence of N-acetyl cysteine [132,146]. Similarly, gefitinib has also been demonstrated to produce a dose-dependent increase in oxidative stress, which has been associated with induced EMT [147,148]. On the contrary, in gefitinib-resistant cells, elevated expression of the antioxidant enzyme peroxiredoxin II resulted in downregulation of ROS and attenuated apoptosis [133]. For that reason, peroxiredoxin II is as a potential target for overcoming gefitinib resistance. 

The strong synergistic antiproliferative and proapoptotic effects of EGFR TKIs and the histone deacetylase inhibitor vorinostat seems to result from induced changes in redox homeostasis. Administration of vorinostat with erlotinib or gefitinib leads to c-Myc downregulation and simultaneous Keap1 upregulation. Furthermore, it was hypothesized that Keap1 mutations leading to inactivation could be used as predictive factors of EGFR TKI resistance [149,150]. Similarly, erlotinib also induces cytotoxicity via NOX4-induced H_2_O_2_ generation, which seems to be reversible after N-acetyl cysteine treatment [151]. The least-studied EGFR-targeted TKI, afatinib, has been associated with increased ROS production, which leads to the development of its resistance [131]. Taken together, development of more specific molecular targets to overcome resistance needs to be confirmed in prospective clinical trials in order to optimally stratify patients for these costly and potentially toxic treatments. 

### 10.3. Free-Radical-Mediated Effects of HER-Targeted Therapy

HER-targeted therapy can be designed either to affect HER1 and HER2 tyrosine kinase receptors or HER2 dimerization. The only TKI approved for treatment in HER2-overexpressing breast cancer patients is lapatinib. Increased ROS levels are observed upon treatment with lapatinib and its analogue, GW583340. As expected, decreased ROS production, together with increased antioxidant capacity (SOD1, SOD2, and GSH), was detected in lapatinib-resistant breast cancer cells. Additionally, a SOD mimic overcame resistance in GW583340-sensitive cells [134]. In patients with resistance to lapatinib, treatment with the covalent JNK inhibitor synergistically causes cell death by reducing transcriptional activity of NFkB, AP1, and Nrf2. Being the master regulators of antioxidant response, their decreased activity induces a 10-fold increase in reactive oxygen species that is cytotoxic to cancer cells and is rescued by the addition of exogenous antioxidants [152]. Trastuzumab is a monoclonal antibody designed to inhibit HER2 dimerization and is approved as a novel neoadjuvant therapy for HER2-positive breast cancer, as well as metastatic breast and gastric cancers. It induces cytotoxicity, inhibiting MAPK and PI3K/Akt pathways [153]. In order to enhance the cytotoxicity potential of trastuzumab, the other HER2 dimerization inhibitor pertuzumab is generated [154], which is indicated to enhance the antiHER efficacy in combination with trastuzumab, both in neoadjuvant and metastatic settings. The combined treatment with these two drugs leads to inhibition of the transcription factor nuclear factor erythroid-derived 2-like 2 (NFE2L2). The most challenging issue regarding trastuzumab treatment is development of resistance, probably due to loss of function of PTEN, a tumor suppressor, resulting from increased Trx1 levels. By binding to PTEN, Trx-1 enables full Akt signaling and affects cell growth, which was confirmed in trastuzumab-resistant cells that regained drug sensitivity after treatment with the Trx-1 inhibitor 1-methylpropyl 2-imidazolyl disulphide (PX-12) [135]. Therefore, in vivo studies with PX-12 aiming to overcome trastuzumab resistance seem reasonable. 

### 10.4. Free-Radical-Mediated Effects of PDGFRα, KIT, ABL, and CSF-1 Receptor-Targeted Therapy

It has been shown that imatinib targets platelet-derived growth factor receptor-α (PDGFR-α), KIT (CD117), as well as nonreceptor tyrosine kinase (ABL) and colony-stimulating factor-1 receptor (CSF-1) [126]. Although the majority of the data explaining ROS-mediated mechanisms of imatinib are obtained from leukemia, ROS-dependent apoptosis has also been reported in melanoma cell lines [102]. 

### 10.5. Free-Radical-Mediated Effects of BRAF-Targeted Therapy

Vemurafenib is designed to target gene-encoding proto-oncogene B-Raf (especially in BRAF V600E melanoma), and thus suppresses the RAS/MEK/ERK signaling pathway. Apart from this specific mechanism of action, vemurafenib stimulates NO and O_2_^·^ production and induces depolarization of mitochondrial membranes, affecting respiration [37,136]. Furthermore, it appears that vemurafenib has other notable and partly ROS-dependent therapeutic effects, independent of BRAF V600E inhibition. Indeed, vemurafenib decreases the metastatic potential of melanoma by inducing the oxidative stress regulator PGC1α and by further lowering expression of numerous integrins [155]. 

## 11. Conclusions

Cancer represents a sort of ontogenetic and phylogenetic regression at the cellular and tissue levels, respectively. During this process, all constituents of the tumor mass actively contribute to its development, progression, and dissemination. Shifting from aerobic to near-anaerobic states, the roles of ROS/RNS in regulation of the main physiological processes related to tumor expansion and spreading become more profound and might affect therapy response. New targeted treatments of solid tumors, such as tyrosine kinase inhibitors and monoclonal antibodies, which have attracted much attention in the past decade, have also been shown to modulate the ROS/RNS balance by increasing the oxidative stress up to a level that overwhelms the antioxidant capacity of cancer cells. Moreover, these treatments influence reactive oxygen and nitrogen species, which function as second messengers, and in this way affect redox signaling. Such agents are designed to target VEGFR, EGFR, HER, BRAF, and PDGFR. Still, in contrast to beneficial effects, some of these agents might increase the antioxidant capacity of cancer cells by upregulating GSH levels, contributing to cancer cell growth and survival. In the future, special attention should be given to the possibility that oxidative and nitrosative stress biomarkers might serve in predicting the therapeutic effects and drug resistance development of these novel targeted treatments.

## Figures and Tables

**Table 1:** Reactive species-mediated effects of receptor-targeted therapy.

Target	Drug Name	Implicated ROS	Suggested Mechanism	Putative Redox Biomarkers	References
**VEGFR**	Axitinib	Not specified	Not specified	Oxidative DNA damage byproducts	[126]
	Sunitinib	↓NO	Increased GSH down regulated NOS	-	[127,128]
	Sorafenib	↑H_2_O_2_, O_2^−^_, NO	Mitochondrial dysfunction and GSH depletion	Advanced oxidation protein products	[129]
**EGFR**	Crizotinib	↑O_2^−^_	Prx upregulation associated with drug resistance	-	[130]
	Afatinib	Not specified	Oxidative stress associated with drug resistance	-	[131]
	Erlotinib	Not specified	Induced ROS-mediated apoptosis	-	[132]
	Gefitinib	Not specified	Prx II upregulation associated with drug resistance	-	[133]
**HER1/HER2**	Lapatinib	Not specified	Upregulated SOD1/ SOD2 and GSH associated with drug resistance	-	[134]
**HER2 dimerization**	Trastuzumab	Not specified	Increased Trx-1 associated with drug resistance	Restoration of plasma antioxidant activity	[135]
**PDGFRα, KIT, ABL, CSF-1 receptor**	Imatinib	Not specified	ROS-dependent apoptosis	-	[126]
**BRAF V600E**	Vemurafenib	↑ NO and O_2^−^_ production	Depolarization of mitochondrial membrane, induced PGC1α	-	[136,137]

JNK = c-Jun N-terminal kinase; Prx II = peroxiredoxin II; HER = human epidermal growth factor receptor; PDGFR = platelet-derived growth factor receptor; KIT (CD117) = proto-oncogene, receptor tyrosine kinase; ABL = non-receptor tyrosine kinase; CSF-1 = colony-stimulating factor-1; PDGFR = platelet-derived growth factor receptor; EGFR = epidermal growth factor receptor; PGC-1 = peroxisome proliferator-activated receptor-gamma coactivator-1; Trx = thioredoxin; GSH = glutathione; VEGFR = vascular endothelial growth factor receptor. ↓ decreased, ↑ increased.

## Abbreviations

ABL = nonreceptor tyrosine kinase	AMPK = AMP-activated protein kinase
AP-1 = activating protein-1	CAF = cancer-associated fibroblast
CAT = catalase	cGMP = cyclin guanosine monophosphate
CREB = cAMP response element-binding protein	CSF-1 = colony-stimulating factor-1
DC = dendritic cell	DR5 = Death receptor 5
EGF = epidermal growth factor	EGFR = epidermal growth factor receptor
EMT = epithelial-mesenchymal transition	FGF = fibroblast growth factor
GCL = glutamate cysteine ligase	GPX = glutathione peroxidase
GSH = glutathione	GST = glutathione S-transferase
HER = human epidermal growth factor receptor	HDAC = Histone deacetylase
HIF-1 = hypoxia-inducible factor 1	JNK = c-Jun N-terminal kinase
Keap1 = kelch-like protein 19	KIT (CD117) = proto-oncogene, receptor tyrosine kinase
MAPK = mitogen activated protein kinase	MDSC = myeloid-derived suppressor cell
MMP = matrix metalloproteinases	NFE2L2 = nuclear factor erythroid-derived 2-like 2
NFkB = Nuclear Factor-κB	NOX = NADPH oxidase
Nrf2 = nuclear factor erythroid 2-related factor 2	PDGF = platelet-derived growth factor
PDGFR = platelet-derived growth factor receptor	PGC-1 = peroxisome proliferator-activated receptor-gamma coactivator-1
PI3K/Akt = phosphoinositide-3-kinase–protein kinase B	PTEN = phosphatidylinositol-3,4,5-trisphosphate 3-phosphatase
PTX = paclitaxel	PX-12-1 = methylpropyl 2-imidazolyl disulphide
RKIP = Raf kinase inhibitor protein	Snail = transcription factor
SOD = superoxide dismutase	TAF = tumor associated fibroblast
TAM = tumor-associated macrophage	TIL = Tumor infiltrated lymphocyte
TKI = tyrosine kinase inhibitor	Trx = thioredoxin
VEGF = vascular endothelial growth factor	YY1 = Yin Yang 1 transcription factor
